# Erbium Probes of Magnetic Order in a van der Waals Material

**DOI:** 10.1021/acs.nanolett.6c03698

**Published:** 2026-10-13

**Authors:** Guadalupe García-Arellano, Gabriel I. López-Morales, Kang Xu, Samra Husremović, Jiayi Li, Arun Ramanathan, Xavier Roy, Cyrus E. Dreyer, Carlos A. Meriles

**Affiliations:** † Department of Physics, CUNY-City College of New York, New York, New York 10031, United States; ‡ CUNY-Graduate Center, New York, New York 10016, United States; § Department of Chemistry, 5798 Columbia University, New York, New York 10027, United States; ∥ Department of Physics and Astronomy, 12301 Stony Brook University, Stony Brook, New York 11794-3800, United States; ⊥ Center for Computational Quantum Physics, Flatiron Institute, 162 fifth Avenue, New York, New York 10010, United States

**Keywords:** telecom photon emission, rare-earth ions, Er^3+^, two-dimensional magnets, CrSBr

## Abstract

Embedded atomic emitters offer a complementary approach to characterizing magnetism in two-dimensional materials by accessing the magnetic environment from within. Here, we demonstrate that ion-implanted Er^3+^ centers act as internal optical probes in the van der Waals antiferromagnet CrSBr. Temperature-dependent telecom photoluminescence reveals signatures of the magnetic transition, persistent local magnetic behavior above the bulk Néel temperature, pronounced thermal hysteresis, and redistribution of optical spectral weight near the transition. Upon cooling, the Er resonances sharpen to the instrumental limit, pointing to remarkably homogeneous local environments despite implantation. Measurements spanning a 20-fold range of implantation fluence, structural characterization, and first-principles calculations suggest that the detected emission originates predominantly from an optically active subset of Er centers occupying well-defined environments and minimally perturbing the host magnetism. These results establish rare-earth defects as embedded probes of local magnetism and open opportunities for quantum-optical interrogation at individual lattice sites.

## Graphical abstract

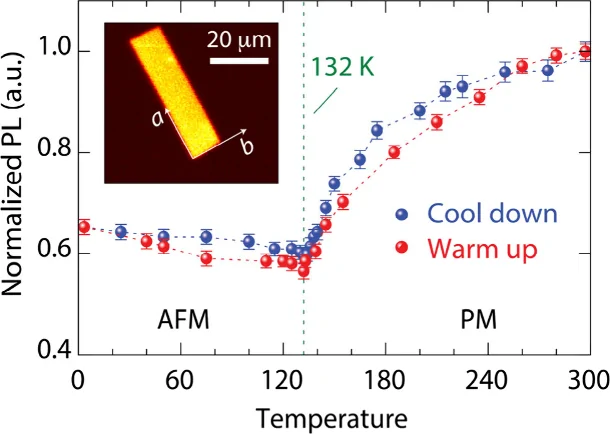

Crystalline van der Waals (vdW) magnets provide a fertile platform to study correlated excitations and engineer hybrid magnonic–optoelectronic devices at the two-dimensional (2D) limit. Among them, the magnetic semiconductor chromium sulfide bromide (CrSBr) is distinctive, as it combines a direct electronic gap in the near-infrared and a comparatively high magnetic ordering temperature;^1^ further, it exhibits quasi-one-dimensional (quasi-1D) electronic character that drives highly anisotropic transport and optics.^2^ In bulk and few-layer form, CrSBr shows A-type antiferromagnetism (AFM) with a Néel temperature *T*_*N*_ ≈ 132 K and large in-plane anisotropy, as well as good exfoliability and air stability, making it an attractive host for exploring mixed 1D/2D physics and magneto-optical coupling.^3,4^ In this mixed-dimensional setting, excitonic resonances track field- and temperature-driven reorganizations of magnetic order,^5–8^ including a 30–40 K ‘hidden-order’ window^3,9^ as well as direct exciton–magnon coupling and tunable hybridization;^10,11^ these magneto-optical contrasts furnish practical readouts of magnetic state in CrSBr.^5,12,13^

Adding to various characterization methods,^4,5,7,14^ prior work leveraged individual nitrogen-vacancy (NV) centers in diamond as atomic probes to image CrSBr magnetism with high spatial resolution.^15^ Complementary ensemble NV *T*_2_ noise magnetometry has quantified critical slowing-down and extracted critical exponents.^16^ These approaches deliver quantitative nanoscale maps of stray fields, magnetic anisotropy, and phase coexistence in monolayer and bilayer samples; however, because the NV is external to the sample, the spatial averaging length is typically set by an NV–sample standoff on the order of tens of nanometers, which inevitably averages magnetic textures over that scale.

As demonstrated recently for Yb:CrI_3_,^17^ an alternative approach is to embed the atomic sensor within the magnetic material. Here, we extend this concept to CrSBr using optically active Er^3+^ defects, which provide telecom-band optical transitions with magnetic-dipole character while sensing their immediate atomic environment. Temperature-dependent measurements reveal signatures of the antiferromagnetic transition, persistent local magnetic behavior above the bulk Néel temperature, pronounced thermal hysteresis, and a redistribution of optical spectral weight near the transition. Upon cooling, the Er resonances progressively sharpen to the instrumental limit, pointing to remarkably homogeneous local environments.

The samples we investigate were produced by exfoliation from a high-purity CrSBr single crystal followed by Er ion implantation (10 keV, 1 × 10^13^ and 5 × 10^11^ ions cm^–2^, respectively denoted high (H) and low (L) dose). From the estimated penetration depth, we estimate an Er concentration of ∼14 and ∼0.7 ppm for the H- and L-dose samples, respectively (Supporting Information (SI), Section I). In our experiments we use a home-built confocal microscope with below-gap laser excitation at 980 nm (Figure 1a, see also SI, Section II). To facilitate detection—especially demanding in time-resolved and spectroscopy measurements, see below—we focus on relatively thick flakes (≥80 nm) where the PL brightness is highest. Figure 1b compiles a reflected-light image, a confocal PL map integrated over the 1.5–1.6 μm band, and the corresponding atomic force microscopy topography with an accompanying line profile. The H-dose flake we present here features a characteristic rectangular geometry, elongated along the *a*-axis. The PL is colocalized with the crystal and follows the flake geometry. In this particular illustration, the emitted PL is homogeneous throughout the crystal, consistent with the uniform thickness of the flake.

**Figure 1:**
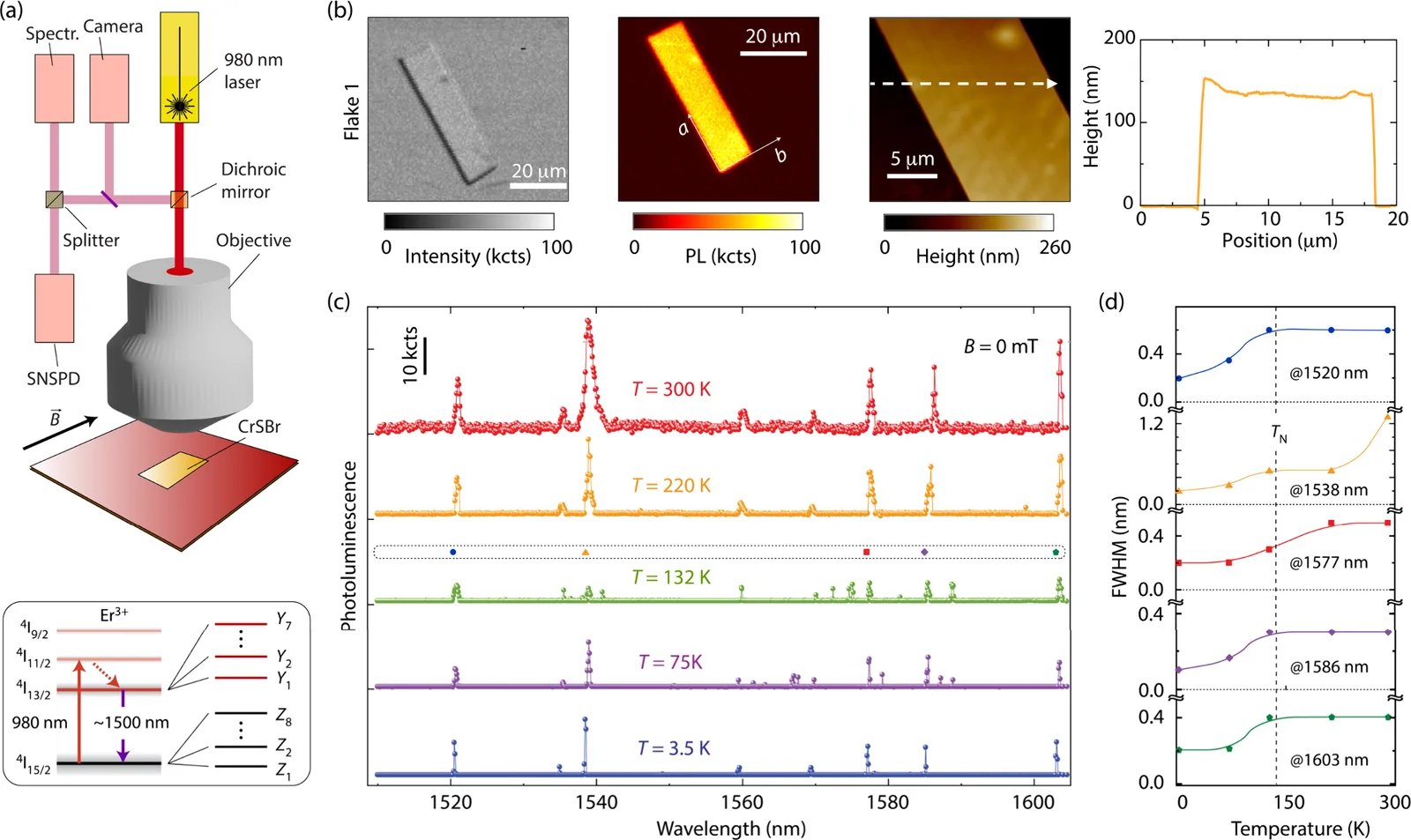
Er telecom emission from Er:CrSBr. (a) Schematic of the experimental setup; a 980 nm laser excites Er^3+^ emitters in CrSBr; telecom-band photons are directed either to a spectrometer (Spectr.) or to a superconducting nanowire single-photon detector (SNSPD). The magnetic field *B⃗* is produced by a permanent magnet (not shown); a cryo-workstation allows us to vary temperature from ambient down to 3.5 K. Bottom inset: Simplified Er^3+^ energy-level diagram showing 980 nm excitation and telecom-band emission near 1.5 μm. (b) Overview of an Er-implanted CrSBr flake (Flake 1, H-dose): Room-temperature reflected-light micrograph (left), confocal PL map integrated in the telecom band (center-left), atomic force microscopy topography (center-right), and height profile along the dashed line (right); *a* and *b* denote the CrSBr crystallographic axes. (c) Telecom-band PL spectra at selected temperatures between 300 and 3.5 K at *B* = 0. Multiple Er^3+^ resonances are observed across the telecom band and sharpen markedly upon cooling, becoming resolution-limited (∼0.1 nm) at low temperature. (d) Temperature dependence of the line width (fwhm) for selected emission lines, each highlighted by an accompanying symbol (dashed rectangle in (c)).

High-resolution spectroscopy (Figure 1c) reveals a discrete set of Er^3+^ resonances spanning the telecom band corresponding to optical transitions between the ^4^ *I*_13/2_ and ^4^ *I*_15/2_ manifolds of Er^3+^ in a crystalline environment.^18^ As the sample is cooled from room temperature, the overall peak amplitudes initially decrease toward the Néel temperature and partly recover thereafter. Concurrently, spectral weight is redistributed among the Er resonances, with new weak transitions appearing as the system approaches and crosses *T*_N_. Importantly, this redistribution does not terminate at the transition: additional resonances become visible upon further cooling to 75 K that are not apparent at 132 K, before the spectrum evolves toward the simpler set of dominant transitions observed at 3.5 K. The continued emergence and disappearance of optical transitions well within the antiferromagnetic phase indicates that the local environment governing the Er optical selection rules continues to evolve below the onset of long-range order.

We presently see two plausible and not mutually exclusive mechanisms. First, changes in the local magnetic order can modify the symmetry and mixing of the Er crystal field states, thereby redistributing oscillator strength among transitions between the ^4^I_13/2_ and ^4^I_15/2_ manifolds. In this picture, resonances that are weak or effectively dark in one magnetic configuration acquire an appreciable radiative strength as the magnetic environment evolves. Second, the local magnetic field generated by the Cr sublattice may shift the Er transition energies themselves, which would allow us to interpret these features as field-displaced resonances. Our present data do not allow us to distinguish unambiguously between these two contributions, although both may occur simultaneously.

A distinct temperature dependence is observed in the line widths (Figure 1d). Upon cooling, the Er resonances progressively narrow, as expected from the suppression of phonon-mediated broadening, with several transitions ultimately reaching the ∼0.1 nm instrumental resolution at 3.5 K. While this narrowing need not itself reflect evolving magnetic order, the exceptionally sharp low-temperature resonances place a stringent upper bound on the spectral heterogeneity of the optically active Er population, pointing to a remarkably well-defined local environment.

We find equally strong signatures of the interplay between CrSBr magnetism and the Er response when we probe the integrated photoluminescence (PL) across the flake. Indeed, cooling the implanted flakes from room temperature reveals a pronounced suppression of Er^3+^ telecom PL that deepens into a clear minimum around *T*_*N*_ (Figures 2a through 2c). Accompanying this change in PL intensity, we also measure a mirrored temperature dependence of the emission lifetime, exhibiting a clear maximum at *T*_*N*_ (Figures 2d and 2e). This anticorrelation between PL intensity and the millisecond-scale lifetimes observed here indicate that the optical response remains highly efficient, i.e., nonradiative recombination is weak.^19,20^

**Figure 2:**
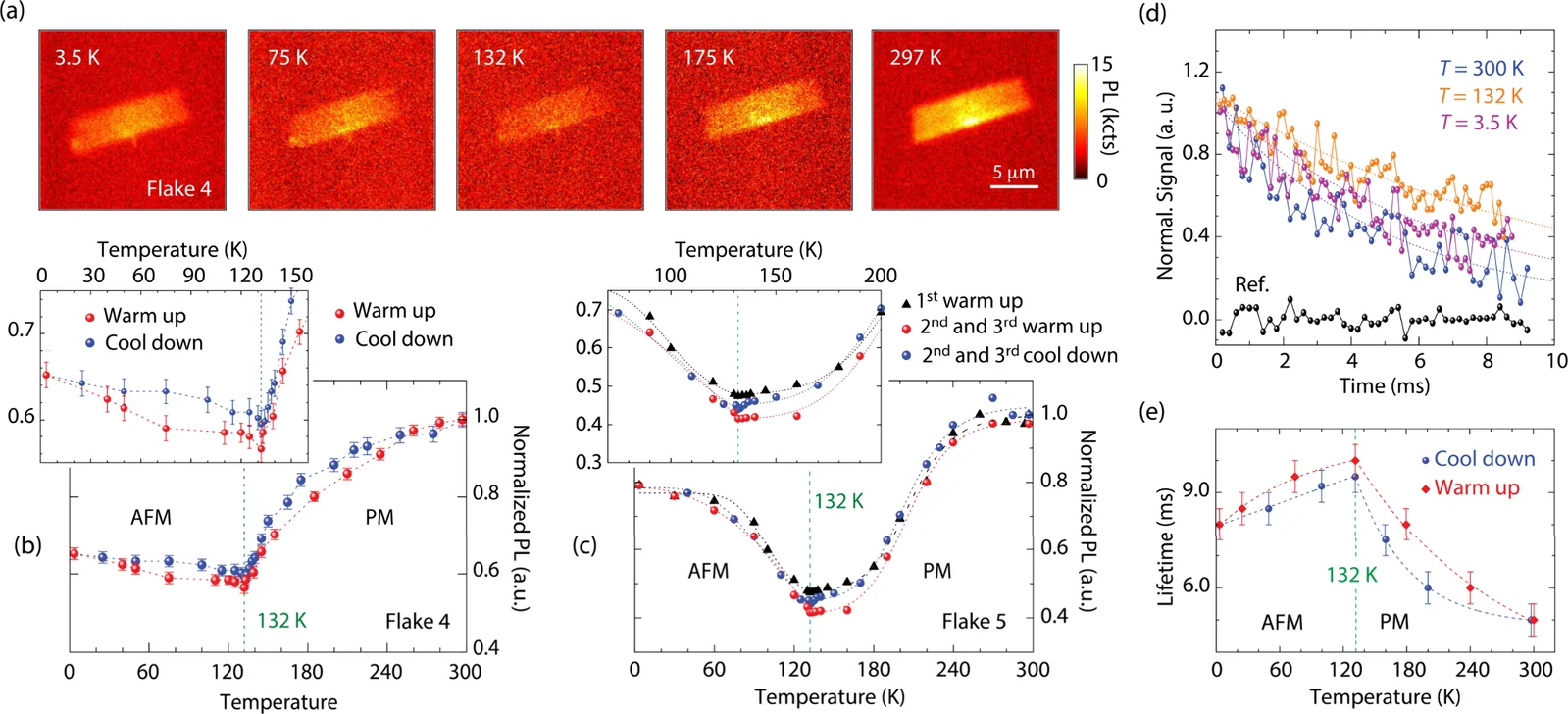
Er^3+^ PL and lifetime across the Néel transition. (a) Telecom-band PL images for Flake 4 (∼70 nm thick) acquired at selected temperatures during a temperature cycle (cooling and reheating, as indicated). The emission weakens markedly near the Néel temperature *T*_*N*_ ≈ 132 K. (b) Flake-integrated PL versus temperature for cooling (blue) and warming (red), revealing pronounced thermal hysteresis with a minimum centered near *T*_*N*_. Inset: magnified view of the 90–160 K range. (c) Normalized PL from repeated cycles on a thicker flake (Flake 5, ∼600 nm), highlighting the convergence and ultimate reproducibility of the dip near *T*_*N*_ and the hysteretic envelope. (d) Representative time-resolved PL traces at several temperatures (symbols) for Flake 4 with single-exponential fits (dashed). The excited-state lifetime increases on approaching *T*_*N*_. A reference trace from an off-Er region is shown in black. (e) The extracted PL lifetime peaks near *T*_*N*_ and decreases away from the transition (arrow indicates sweep direction). No external magnetic field is present throughout these measurements.

We note this anticorrelated PL/lifetime response and the redistribution of spectral weight observed in Figure 1c may be related, since both are expected to depend on how the local magnetic environment modifies the Er crystal-field states and their optical transition strengths. However, while the integrated PL and lifetime become comparatively stable well below *T*_*N*_, the individual spectral features continue to evolve to at least 75 K. This suggests that the two observables may probe the same underlying magnetic reorganization at different sensitivities.

The sharpness of the optical resonances and the observation of a seemingly unperturbed Néel transition—prone to temperature shifts even in the presence of comparatively low strain^21–23^ —are difficult to reconcile with the notion of implantation-induced damage, necessarily associated with a broad distribution of local environments. Er centers in distorted lattices, however, are known to display efficient nonradiative relaxation channels, leading us to hypothesize that the PL we measure originates from a rather reduced, protected subset of emitters subject to minimal lattice disorder (see below). This interpretation is consistent with our measurements in the L-dose flakes, displaying temperature-dependent spectroscopy and PL virtually identical to Figures 1 and 2—including absolute PL brightness—despite the 20-fold reduction in the implantation fluence, and even after 400 °C annealing (SI, Section III). Interestingly, an extreme manifestation of this “self-selectivity” effect is provided by the surrounding amorphous SiO_2_ substrate, which receives the same Er implantation fluence as the CrSBr flakes yet remains essentially dark. Lastly, we note that transmission electron microscopy (TEM) following implantation (SI, Section I) supports the notion of a highly crystalline CrSBr lattice, although the inherent locality of the implantation damage and the relative scarcity of emitters—featuring concentrations 3 to 4 orders of magnitude lower than in preceding studies of rare-earth-doped van der Waals magnetic materials^17^—did not allow us to study individual Er sites.

Viewed through this lens, our results suggest a rich temperature dependence of the local magnetic order. At *T*_*N*_, interlayer phase locking is weakest and the crystal hosts short-correlated AFM fragments and mobile domain walls;^4,7,9,15,24^ correspondingly, incomplete sublattice cancellation and wall/edge/defect moments yield the largest net local field, producing the pronounced PL minimum (and lifetime maximum). Below *T*_*N*_, long-range AFM order gradually strengthens and sublattice cancellation at the Er site improves; domain walls freeze and the local field decreases, so the emission rate and PL increase. This recovery is more pronounced in thicker flakes (∼600-nm-thick Flake 5 in Figure 2c), suggesting the order improves as flakes thicken to become bulk crystal material (we have observed similar thickness-dependent PL trends when comparing the response from different areas in nonuniform flakes, see SI, Section III).

The above interpretation assumes that the Er PL is governed by the local magnetic field itself, not the temperature, thus implying a magneto-optic response for Er. Independent room-temperature measurements under an externally applied in-plane magnetic field show that a field of only 0.3 T produces a pronounced (∼20%) reduction of the Er PL together with a corresponding increase in the excited-state lifetime (SI, Section III). This is precisely the anticorrelated behavior observed upon approaching *T*_*N*_ and lends support to the notion of an optical response governed primarily by the local magnetic field experienced by the Er ions, with temperature acting indirectly through its influence on the magnetic state of the host. Using the PL dependence in Figure 2 as a rough proxy, the fractional dimming at *T*_*N*_ corresponds to an effective in-plane field *B*_*eff*_ of a few hundred mT at the Er site; we emphasize this is a phenomenological equivalence, not a direct measurement of the microscopic dipolar field. Remarkably, reduced PL intensity persists over a broad temperature range *above T*_*N*_, indicating residual local magnetic fields or short-range order sensed at the defect site; the high temperature shoulder extends further in the thicker flakes, consistent with longer correlation lengths and greater metastability.^3–5,25,26^

It is worth contrasting this response with prior NV-based studies—both single-spin scanning magnetometry^15^ and ensemble *T*_2_ noise spectroscopy^16^—where the sensed stray fields are orders of magnitude weaker and critical behavior comparatively concentrates near the transition temperature. We attribute these differences to the external nature of these sensors, necessarily averaging over nanometric stand-off distances. For example, in scanning NV experiments on CrSBr, the effective NV-sample spacing that sets spatial resolution is greater than ∼10 nm, which inherently smooths magnetic textures on that scale; Er, by contrast, is an internal, atomic-site probe that reports on fields within a few ångströms of its lattice position, a sensing locality akin to muon spin relaxation (μSR) spectroscopy.^9^

A separate hallmark in the results of Figure 2 is the thermal hysteresis observed on cycling the temperature, in both the PL intensity and lifetime. When warming from low *T* (red dots in Figure 3b), the PL remains comparatively dimmer than on cooling at the same temperature (blue dots), suggesting persisting magnetic order upon warming, i.e., a larger remnant order parameter carried upward from the AFM phase, before relaxing toward the paramagnetic state. A further nuance is that the hysteresis trains before it settles. In the thicker flake of Figure 2c, for example, the first warm-up (black triangles) defines a baseline; the subsequent cool-down (blue circles) then lies below that first warm-up, thus displaying the opposite trend from Figure 2b, where cool-down curves sit above warm-up curves at the same *T*. Only after additional cycles (second warm-up → third cool-down → third warm-up) do the traces converge to a repeatable loop with the standard ordering (cool-down above warm-up) observed in Figure 2b. This history dependence points to slow domain/wall reconfiguration and pinning–depinning that is especially pronounced in thicker flakes; repeated thermal excursions anneal the metastable landscape, after which the system reproducibly follows the same microscopic pathway through the antiferro- to paramagnetic transition.

**Figure 3:**
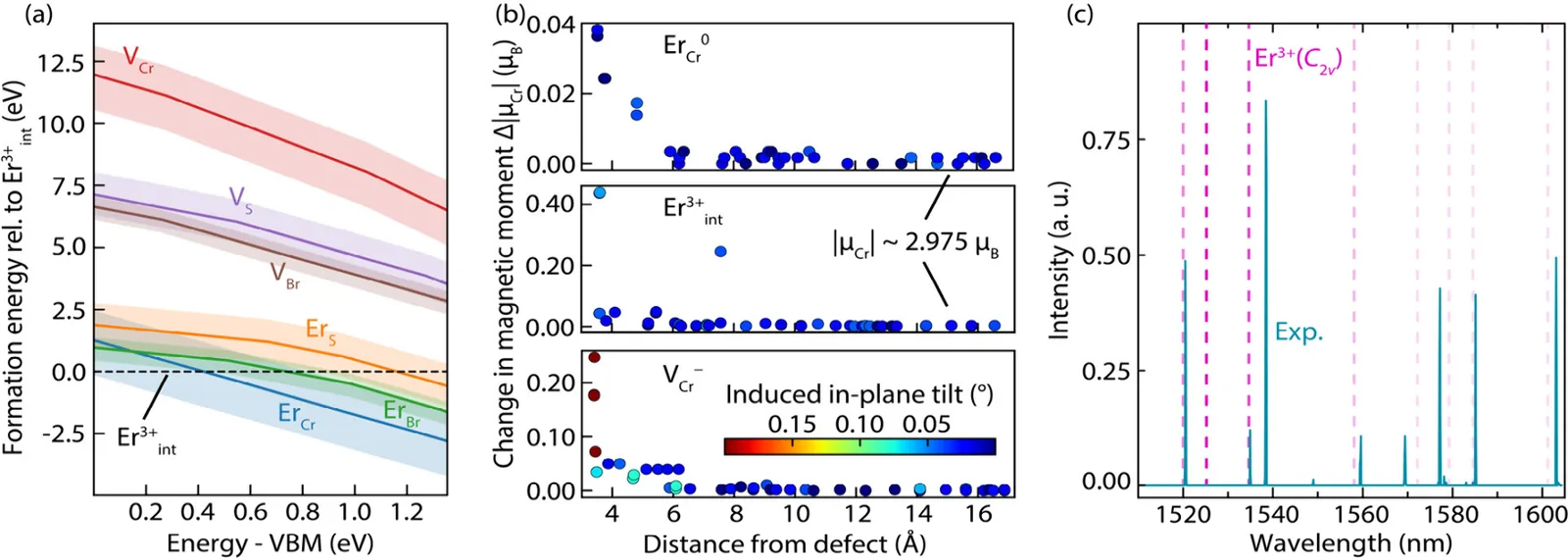
First-principles modeling of Er^3+^ dopants in bulk CrSBr. (a) Defect formation energies for Er^3+^ in S, Br, and Cr sites, referenced to the interstitial Er^3+^ case (i.e., between layers). Isolated native vacancies are included for comparison. Color-coded shaded regions denote the range of chemical potentials corresponding to element-rich and element-poor growth conditions. (b) Change in the magnitude of Cr magnetic moments as a function of distance away from a substitutional Er (top), interstitial Er (center), and Cr vacancy (bottom), all in their energetically favored charge states. The scatter color scale represents in-plane tilt of the magnetic moments with respect to the pristine CrSBr case. Here, the magnetic moment quantization axis is chosen to point along the easy-axis of CrSBr (*b*-axis). (c) Calculated Er^3+^ optical transition wavelengths for a substitutional Er_Cr_ center under *C*_2*v*_ symmetry obtained from a semiempirical Slater–Hund many-body model (vertical dashed lines), compared with the experimental PL spectrum at *T* = 3.5 K (solid blue curve). The calculations include thermal population of the excited-state manifold; relative line strengths are encoded in the level of transparency.

To gain microscopic insight into the nature of the implanted Er centers, we performed first-principles calculations based on density functional theory with on-site Hubbard corrections (DFT+*U*) to consider candidate substitutional and interstitial configurations together with their charge-state stability (SI, Section IV). As summarized in Figure 3a, Er substituting for Cr (Er_Cr_) is consistently the energetically preferred defect over a broad range of chemical potentials and naturally adopts the neutral charge state, reflecting the matching oxidation states of Er^3+^ and Cr^3+^.

The calculations provide a rationale for the remarkable spectral homogeneity observed experimentally: because the crystal-field levels of trivalent lanthanides are highly sensitive to local symmetry and charge balance, the pronounced differences in the stability of the various defect configurations imply that only a limited subset of Er centers remain optically active. In this light, the energetic preference for substitutional Er_Cr_ rationalizes the emergence of a well-defined set of narrow optical resonances, despite the stochastic nature of the implantation process.

Along the same lines, the calculations further indicate that the Er magnetic moment has only a weak influence on the surrounding Cr magnetic sublattice. As captured in Figure 3b, changes in the Cr magnetic moments across energetically favored defect configurations remain localized around the defect center. Furthermore, neighboring Cr moments retain their intrinsic intralayer ferromagnetic and interlayer antiferromagnetic ordering (with negligible in-plane tilts), changing in magnitude by at most ∼13% (for the interstitial Er case). Interestingly, our results show the substitutional (Er_Cr_) defect introduces the smallest effects, even compared to the plain Cr vacancy (V_Cr_). This weak perturbation likely reflects the highly localized nature of the Er 4*f* electrons together with the matching magnetic moments of Er^3+^ and Cr^3+^.

We constructed an effective correlated many-body model for the Er^3+^ crystal-field states under *C*_2*v*_ symmetry, the point group associated with substitutional Cr sites in bulk CrSBr. The crystal-field splitting and doublet ordering were obtained by fitting the measured spectrum, accounting for the thermal population of the excited-state manifold at 3.5 K. Owing to the strong magnetic interactions in CrSBr, our present ab initio framework cannot reliably capture the electric components of the transition dipole moments, which depend sensitively on hybridization between the Er 4*f* states and the host lattice.^27,28^ The magnetic components of the transition dipole moments, however, are well-defined within the many-body basis and provide direct access to the magnetic character of the optical transitions.

The model predicts that most of the magnetic oscillator strength is concentrated within the 1520–1540 nm spectral window, distributed among approximately three ^4^ *I*_13/2_ ↔ ^4^ *I*_15/2_ lowest-doublet transitions, consistent with previous studies of Er-implanted materials.^18,29^ On the other hand, the agreement with the observed spectrum is moderate, which might indicate the importance of the electrical dipole contribution, activated by the Er/bulk hybridization. In terms of the energies of the transitions, it is possible that the disagreements indicate subensembles of Er centers with different crystal fields. However, we note that the small energy scales of the splittings and the relatively low site symmetry (which results in four interdependent tunable parameters) make the theoretical fitting a challenge.

Further, although the Zeeman shifts of the Er crystal field states are expected to be small at the experimental magnetic fields, the dense manifold of near-degenerate doublets allows magnetic-field-induced mixing of the many-body states, thereby modifying the transition dipole moments and hence the PL intensity. A similar mechanism was recently demonstrated for Er:WS_2_ using an effective parametrization by DFT and quantum-embedding calculations, where the field was shown to quench the transition dipole moments and rotate their polarization.^20^ Analogous embedding calculations are presently impractical for CrSBr because of the magnetic nature of the host, but the qualitative correspondence of the experimental observations suggests that the same microscopic mechanism may govern the Er optical response in CrSBr.

The magnetic coupling experienced by Er can be placed on a useful quantitative scale by comparison with Yb:CrI_3_, where preceding work reported an exchange-driven molecular field of order 10 T at substitutional Yb sites, accompanied by pronounced magnetic shifts of the optical transitions.^17^ Such strong coupling was attributed to appreciable Yb–I covalency, which facilitates superexchange between the Yb 4*f* states and the surrounding Cr lattice. Our calculations in CrSBr instead indicate a weaker covalent, ligand-like environment for Er^3+^, suggesting substantially weaker exchange coupling. This expectation is consistent with the spectroscopy: no comparable magnetic shifts or splittings are observed, with several Er resonances instead narrowing to our ∼0.1 nm instrumental resolution of ∼13 GHz near 1.5 μm. While this resolution does not provide a model-independent bound on the exchange field, it disfavors the strong-exchange limit exemplified by Yb:CrI_3_. At the same time, much smaller magnetic fields are sufficient to perturb the Er response (SM, Section IIIa), hence suggesting the observed temperature dependence may arise in a regime where relatively weak local exchange and uncompensated dipolar fields associated with the evolving magnetic texture are both relevant.

This picture also provides a spatial perspective on the magnetic environment sensed by Er. Small-angle neutron scattering measurements^30^ show that intralayer correlations develop below ∼200 K and extend over ∼3 nm near *T*_*N*_. The close correspondence between this evolution and the Er PL suppression supports sensitivity to the finite correlated regions and their associated uncompensated dipolar fields. The somewhat faster decrease of the PL likely indicates a local exchange contribution that develops before correlations extend over several nanometers.

In summary, this work establishes Er^3+^ defects as embedded optical probes of local magnetism in the layered antiferromagnet CrSBr. By combining temperature-dependent telecom spectroscopy, photoluminescence imaging, lifetime measurements, and first-principles modeling, we show that the Er response directly reflects the evolution of the host magnetic state while remaining only weakly perturbative to the surrounding lattice. Besides reproducing the bulk Néel transition, the embedded probes reveal a continuous evolution of the local magnetic environment throughout the ordered phase, signatures of magnetic correlations extending above the bulk transition temperature, and pronounced thermal hysteresis indicative of slow nanoscale magnetic dynamics.

Interestingly, the Er PL remains comparatively flat across 30–40 K, with no distinct anomaly. This behavior suggests that the intrinsic low-temperature crossover reported in CrSBr^9^—often described as a hidden-order/dynamic magnetic regime—does not substantially modify the net local field at the Er site, implying that near-site sublattice cancellation is largely preserved through this range (i.e., the local order sensed by Er remains effectively unchanged).

The demonstration that Er^3+^ ions can function as local optical probes of magnetism in CrSBr establishes the basis for future experiments, for example relying on low-energy implantation to examine few-layer flakes,^31^ resorting to photoluminescence spectroscopy (PLE) to resolve Zeeman or hyperfine shifts in the optical transitions, or employing isolated cavity-coupled emitters to achieve single-ion readout.^32,33^ The latter is particularly intriguing as such a site-resolved platform would help pin down the Er lattice position(s) and symmetry while providing direct access to the local magnetic environment, thus enabling measurements that are inaccessible to spatially averaged probes. Similarly, because Er also offers a telecom-compatible spin qubit, integrating coherent control with microcavities could yield on-chip, fiber-ready readout of layered magnets and potentially enable spin–photon–magnon interfaces^34^ to link magnetic excitations in CrSBr to long-distance optical networks.

## Supplementary Material



## Notes

Details on the experimental setup, measurement protocols and conditions, as well as optical spectroscopy and lifetime measurements under cryogenic conditions (PDF)

## Notes

The data that support the findings of this study are available from the corresponding author upon reasonable request.
