# Supplementary material for: Erbium Probes of Magnetic Order in a van der Waals Material
Source: Nano Lett. 2026 Oct 13;26(42):14693–9. doi: 10.1021/acs.nanolett.6c03698 (PMC13721145; doi:10.1021/acs.nanolett.6c03698)
Supplement: Supplementary file 1 [file nl6c03698_si_001.pdf]

Supplementary Information for

Erbium Probes of Magnetic Order in a van der Waals Material

Guadalupe García-Arellano[1], Gabriel I. López-Morales[4], Kang Xu[1], Samra Husremović[3], Jiayi Li[3], Arun Ramanathan[3], Xavier Roy[3], Cyrus E. Dreyer[4,5], and Carlos A. Meriles[1,2,*]

[1]Department of Physics, CUNY-City College of New York, New York, New York 10031, USA.
[2]CUNY-Graduate Center, New York, New York 10016, USA.
[3]Department of Chemistry, Columbia University, New York, New York 10027, USA.
[4]Department of Physics and Astronomy, Stony Brook University, Stony Brook, New York, 11794-3800, USA.
[5]Center for Computational Quantum Physics, Flatiron Institute, 162 5th Avenue, New York, New York 10010, USA.

Content

I. Sample characterization
- *I.a Synthesis of bulk CrSBr crystals*
- *I.b Sample fabrication and implantation details*
- *I.c Transmission electron microscopy of Er-implanted CrSBr flakes*
- *I.d The effect of mild thermal annealing*
- *I.e Correlation between photoluminescence and flake topography*

II Experimental setup
- *II.a Room-temperature optics*
- *II.b Low-temperature operation*
- *II.c Optical spectroscopy and lifetime measurements*

III Extended data sets
- *III.a Field-dependent Er PL*
- *III.b Excitation and emission polarization*
- *III.c PL dependence on flake thickness*
- *III.d Low-fluence implantation control*

IV First-principles calculations
- *IV.a Methods*
- *IV.b Correlated effective model*

I. Sample characterization

I.a Synthesis of bulk CrSBr crystals

The synthesis follows a previously established procedure[1]. The following reagents were used as received unless otherwise stated: chromium powder (99.94%, −200 mesh, Alfa Aesar), sulfur pieces (99.9995%, Alfa Aesar), bromine (99.99%, Aldrich) and chromium dichloride (anhydrous, 99.9%, Strem Chemicals). To begin, $CrBr_3$ was synthesized from Cr (1.78 g, 34.2 mmol) and Br_2 (8.41 g, 52.6 mmol) with one end of the tube held at 1,000 °C and the other end at 50 °C with a water bath. Of note, one end of the tube must be maintained below 120 °C to prevent the tube from exploding from bromine overpressure. Chromium (0.174 g, 3.35 mmol), sulfur (0.196 g, 6.11 mmol) and CrBr3 (0.803 g, 2.75 mmol) were loaded into a 12.7-mm-outer-diameter, 10.5-mm-inner-diameter fused silica tube. The tube was evacuated to ~30 mTorr and flame sealed to a length of 20 cm. It was then placed into a computer-controlled, two-zone, tube furnace. The source side was heated to 850 °C over 24 h, allowed to soak for 24 h, heated to 950 °C over 12 h, allowed to soak for 48 h and then cooled to ambient temperature over 6 h. The sink side was heated to 950 °C over 24 h, allowed to soak for 24 h, heated to 850 °C over 12 h, allowed to soak for 48 h and then cooled to ambient temperature over 6 h. The crystals were cleaned by soaking in a 1 mg ml^{-1} $CrCl_2$ aqueous solution for 1 h at ambient temperature. After soaking, the solution was decanted, and the crystals were thoroughly rinsed with deionized water and acetone. Residual sulfur residue was removed by washing with warm toluene.

I.b Sample fabrication and implantation details

Flakes were prepared by mechanically exfoliating high-purity CrSBr onto 285-nm SiO_2/Si (HF-cleaned just prior to transfer) and screened in bright-field reflection. All flakes were implanted simultaneously in a single broad-beam step (Cutting Edge Ions) at low current density, using 10 keV at 7 deg from the normal to the surface to avoid channeling, with nominal fluences of ~1×10^{13} cm^{-2} and ~5×10^{11} cm^{-2}; we discuss the results from lower-dose samples separately in Section III.d. After implantation, samples were optionally annealed (see below), and mounted in the cryo-workstation with a low-fluorescence, vacuum-compatible resist; room-temperature PL imaging confirmed Er activation, and regions with obvious dose nonuniformity were excluded from quantitative analysis.

To estimate the dopant depth, we first used SRIM (Stopping and Range of Ions in Matter), the community's go-to package for ion-range calculations; for CrSBr ($\rho \approx 4.3 - 4.5$ g cm^{-3}) SRIM predicts a shallow projected range, with average depth of only ~8.5 nm and straggle ≲20 nm (Supplementary Fig. 1a). However, because SRIM treats the target as static, it is ill-suited for heavy ions in heavy, layered substrates, where sputtering, cascade mixing, densification, and stoichiometry evolution during implantation can be significant. In particular, for thin targets and mid-to-heavy ions (approximately $29 < Z < 83$), experiments show that SRIM often underestimates penetration depth, largely due to inaccuracies in electronic stopping and the neglect of channeling[2-5]. The factor of underestimation can range from 2 (as reported for erbium-implanted silicon films[6]) to 10 (as reported in Xe, Ar, N, and O ions implanted in tungsten[7]).

Alternative binary-collision approximation Monte Carlo transport codes—such as IRADINA[8] and TRI3DYN[9]—have been developed to more accurately predict penetration depths. Supplementary Fig. 1b shows the results for TRIDYN, a variant of TRI3DYN that also includes effects like sputtering and evolving target composition, but is better adapted to thicker flakes and is computationally more efficient. We find that TRIDYN shifts the Er distribution markedly deeper (mean ≈10^2 nm) and broadens it (Supplementary Fig. 1b). An experimental cross-check — room-temperature telecom PL versus thickness — rises and then saturates across flakes, showing characteristic thickness of about ~100 nm (Supplementary Fig. 1c). The latter is difficult to reconcile with the shallow SRIM profile, so we adopt the TRIDYN-like distribution as our working estimate.

Finally, we note that 10 keV was the minimum energy available from the implanter, yet this energy is already too large for few-layer CrSBr, for which ions would largely traverse the crystal; accordingly, our study focuses on thicker flakes ($\gtrsim$ 40 nm), where implanted Er remains within the host.

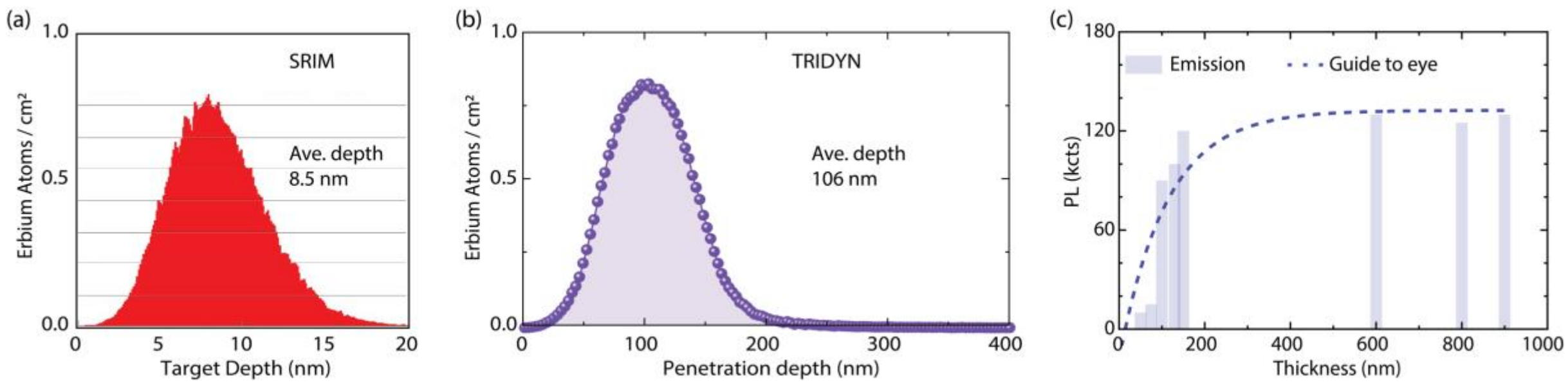

Supplementary Figure 1: Thickness dependence and implantation depth profiles. (a) SRIM simulation of Er penetration for implantation conditions: The projected depth distribution peaks within the top tens of nanometers (average depth ≈ 8.5 nm). (b) TRIDYN simulation for the conditions in (a), yielding a deeper projected range with average penetration depth ≈ 106 nm. (c) Room-temperature telecom PL (integrated 1.50–1.60 µm) versus thickness for representative flakes; bars show the mean PL from each flake, and the dashed line is a guide to the eye. The rise and eventual saturation of brightness with increasing flake thickness is qualitatively consistent with the simulations in (b).

I.c Transmission electron microscopy of Er-implanted CrSBr flakes

Flakes of CrSBr that were previously irradiated with Er were transferred from the initial, electron-opaque SiO_2/Si substrate to the electron-transparent Si_3N_4 holey grid (Norcada) using poly(dimethylsiloxane) (PDMS) transfer. Specifically, a large (1×1 cm) PDMS square that covered the whole chip was pressed onto it and released at room temperature. Next, we found suitable thin flakes on this PDMS for TEM imaging and cut it into a smaller square of approximately 3×3 mm. This PDMS stamp was stuck onto a glass slide, and then a micromanipulator transfer stage (HQ Graphene) was used under inert conditions and without heating to drop the flakes onto the Si_3N_4 holey grid. The grid was cleaned with O_2 plasma for 5 min. immediately before this transfer.

Transmittion electron microscopy (TEM) of the CrSBr flakes was conducted in an FEI Talos F200X microscope operating at 200 kV. For selected area electron diffraction (SAED), a 40 µm aperture was used

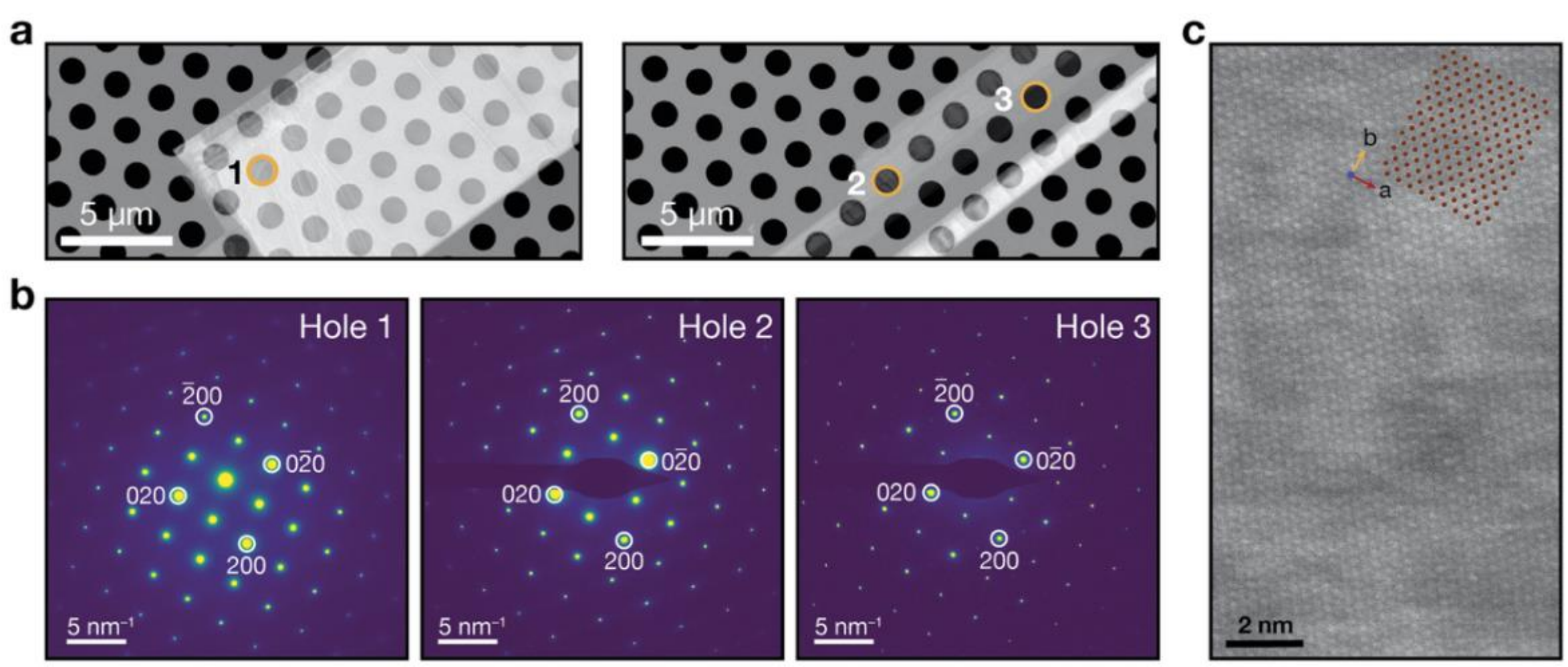

Supplementary Figure 2: Transmission electron microscopy of Er-irradiated CrSBr. (a) High-angle annular dark-field scanning transmission electron microscopy (HAADF-STEM) micrographs of CrSBr flakes irradiated with Er. Imaged regions 1–3 are labeled. (b) Selected-area electron diffraction (SAED) of regions 1–3. The thickness of CrSBr decreases from area 1 to 3, as evidenced by their decreasing HAADF-STEM contrast. (c) Atomic-resolution HAADF-STEM image of Er-irradiated CrSBr acquired from a few-layer region. The image contrast is dominated by the heavy Br atoms, whose crystallographic positions are overlaid on the micrograph.

to isolate an illuminated sample area roughly 700 nm in diameter. High-angle annular dark-field scanning transmission electron microscopy (HAADF-STEM) imaging was performed on the same system at 200 kV with a convergence semi-angle of 10.5 mrad.

Diffraction patterns of Er-irradiated CrSBr flakes of varying thickness all exhibit sharp diffraction peaks, indicating that the irradiated samples retain a high degree of crystallinity (Supplementary Figs. 2a and 2b). The absence of amorphous rings or significant peak streaking further suggests that irradiation-induced amorphization is negligible. Preservation of the crystal structure is also evident in atomic-resolution HAADF-STEM images, which reveal an atomically ordered CrSBr lattice (Supplementary Fig. 2c).

Individual implanted Er ions were not directly identified. This is likely due to several factors, including the relatively low implantation dose, and the limited spatial resolution of the non-aberration-corrected TEM. Nevertheless, the diffraction and STEM data consistently demonstrate that CrSBr retains its crystallinity over a range of sample thicknesses comparable to those used in the optical measurements, indicating that the irradiation process does not significantly degrade the crystal structure.

I.d The effect of mild thermal annealing

The flakes analyzed in the main text were *not annealed*: Initial telecom PL was already strong, optimal conditions for CrSBr were uncertain, and high-temperature holds risk of perturbing the magnetic properties. Nonetheless, to benchmark possible benefits/costs, we performed separate anneals on a subset of flakes (argon atmosphere, 1-h hold, Suppl. Fig. 3a). We find a modest PL increase for mild end-temperatures (180 °C), whereas 400 °C (or higher) generally reduces the PL (Suppl. Fig. 3b). Spectroscopically, annealing does not narrow the Er emission lines, indicating limited improvement in local heterogeneity; further, ~400 °C anneals introduce additional weak lines in the telecom spectrum (Suppl. Fig. 3c).

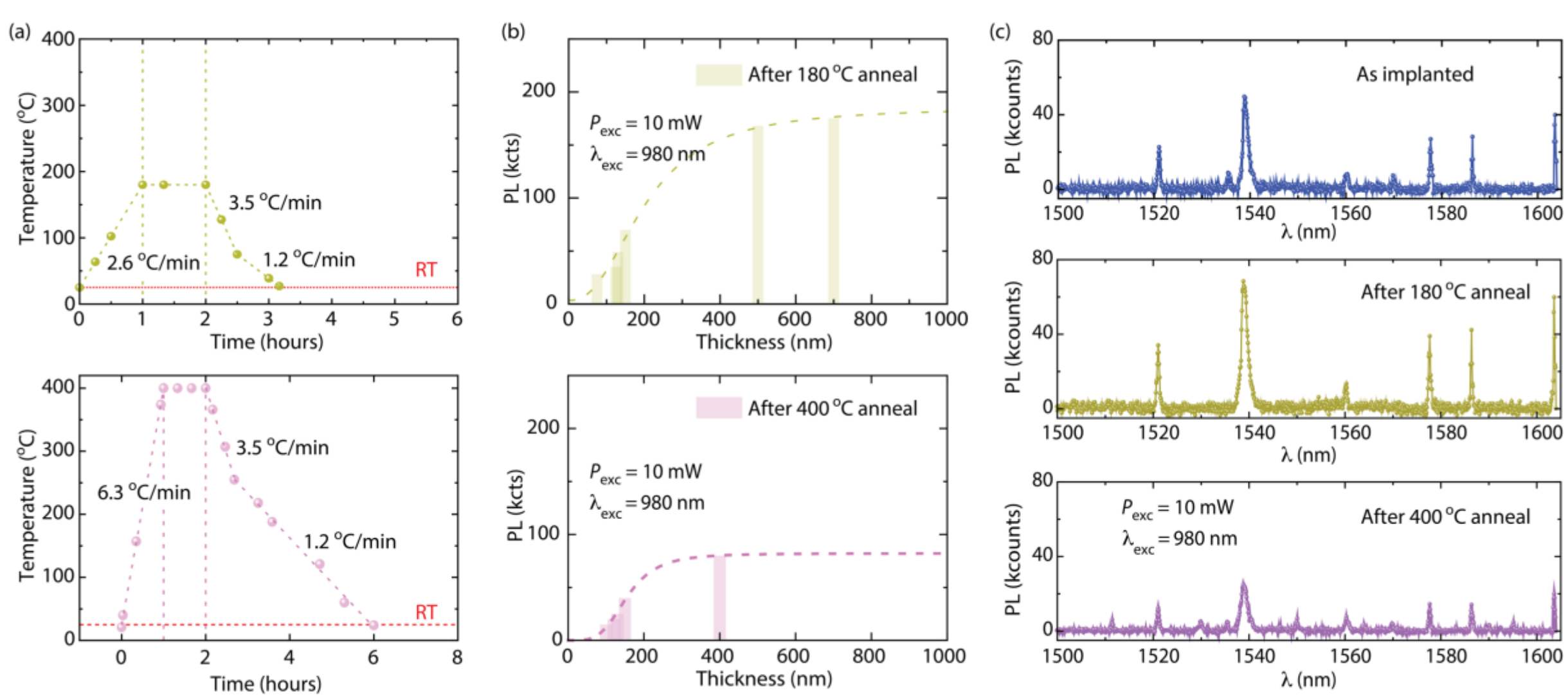

Supplementary Figure 3: Annealing protocols, thickness dependence, and spectral changes of Er:CrSBr. (a) Temperature profiles in argon. Top: 180 °C process with ramp ≈ 2.6 °C min^{-1}, 1 h dwell, and cooldown ≈ 1.2 °C min^{-1}. Bottom: 400 °C process with ramp ≈ 6.3 °C min^{-1}, 1 h dwell, and cooldown ≈ 1.2 °C min^{-1}. (b) Room-temperature telecom PL versus flake thickness (980-nm, 10 mW excitation) measured after each anneal. A mild anneal at 180 °C increases the PL while preserving the thickness trend (dashed guide to the eye), whereas a 400 °C anneal markedly suppresses the signal across devices. (c) Representative room temperature spectra: as-implanted (top), after 180 °C (middle; brighter lines with similar linewidths), and after 400 °C (bottom; overall quenching and appearance of additional weak lines). Gentle annealing leads to mild improvement, while higher temperature processing modifies local environments/defect complexes and degrades emission.

These observations align with two considerations. First, low-temperature activation of implanted Er has precedent in our Er:WS_2 work, where a gentle anneal "turns on" telecom PL without dramatic linewidth changes (i.e., repairs implantation damage and reconfigures nearby vacancies without blanket reordering[10]). On the other hand, CrSBr's layered, halide-containing lattice is susceptible to stoichiometry drift and defect complex formation at elevated temperature; high-T processing can generate secondary phases and alter local crystal fields[11,12], both of which would diminish brightness and spawn new spectral features rather than sharpen existing ones.

A conservative interpretation of the extra peaks after ~400 °C is the emergence of new Er environments, e.g., Er coupled to halogen/chalcogen vacancies or other implantation-induced complexes, charge-state rebalancing at the site, or incipient diffusion/aggregation inside the flake. Disambiguating these scenarios will require systematic anneal series (time/temperature/ambient), compositional probes (e.g., EDS/EELS, ToF-SIMS), and structure-sensitive spectroscopy (site-selective PL, polarization-resolved PLE). For the purposes of this study — focused on magneto-PL sensing — we therefore avoid post-implant anneals and use non-annealed flakes for the main results.

I.e Correlation between photoluminescence and flake topography

Atomic force microscopy (AFM) scans were acquired in tapping mode under ambient conditions (Bruker Fastscan) using Si tips (TESPA-150 BRUKER) with nominal radius < 8 nm. Height and phase channels were recorded simultaneously. Flake thicknesses were obtained from step-height profiles at naturally cleaved edges and/or shallow scratches made outside optical regions of interest. Representative AFM maps and line-cuts are compiled in Supplementary Fig. 4 along with the corresponding optical and PL images (integration window 1.50–1.60 μm, 980 nm excitation) of all flakes presented in the main text.

Within flakes, local PL variations tend to correlate with topographic features: We observed enhanced PL near sharp, protruding edges and inner ridges/wrinkles, possibly because of better out-coupling, as discussed in the main text. Conversely, the PL is more homogeneous in uniform interiors.

II Experimental setup

II.a Room-temperature optics

A home-built confocal microscope is driven by a 980-nm diode laser (single-mode fiber–coupled; linewidth < 0.1 nm). The beam is cleaned by a short-pass/neutral-density stack, expanded to fill the back aperture of a high-NA objective (50×, NA = 0.8 air). Typical powers at the sample are 4 mW unless otherwise noted; operation is in the linear (unsaturated) regime. Back-scattered/emitted light is routed through a Thorlabs DMLP-1138 long-pass mirror, two 1500-nm and one 1250-nm long-pass filters to reject the pump. Detection is either (i) an InGaAs camera behind a 300 mm grating spectrometer (900 grooves/mm blazed at 1.6 μm), or (ii) a fiber-coupled ID Quantique superconducting nanowire single photon detector (SNSPD, system jitter ~60 ps; dark counts < 50 s^{-1}). A rotatable half-wave plate (HWP) sets the pump polarization; a rotatable analyzer (HWP+polarizer) selects the detection polarization. The sample sits on a motorized *xyz* stage for coarse positioning.

II.b Low-temperature operation

A closed-cycle cryo-workstation (Montana Instruments, optical access, vacuum < 10^{-5} Torr) provides temperature control from 300 K to ~3.5 K (stability ±0.1–0.2 K). The sample is mounted on a low-fluorescence copper chuck with silver paint. Temperature is measured at the sample stage (silicon diode) and cross-checked at the cold finger; reported values refer to the stage sensor. A permanent magnet mounted on a kinematic arm provides a static in-plane field (up to ~0.3 T). Field magnitude/direction are calibrated once per session by Hall probe; out-of-plane alignment is verified to within ±3–5°. Unless stated, fields are in-plane. Temperature-dependent measurements were acquired using a step-and-equilibrate protocol rather than a continuous sweep. At each setpoint, the sample was allowed to stabilize for 30 min before confocal acquisition (~3 min per image). The measured temperature drift settled to ~20 mK within 5–10 min, and control measurements showed no difference between 20-min and 12-h stabilization times.

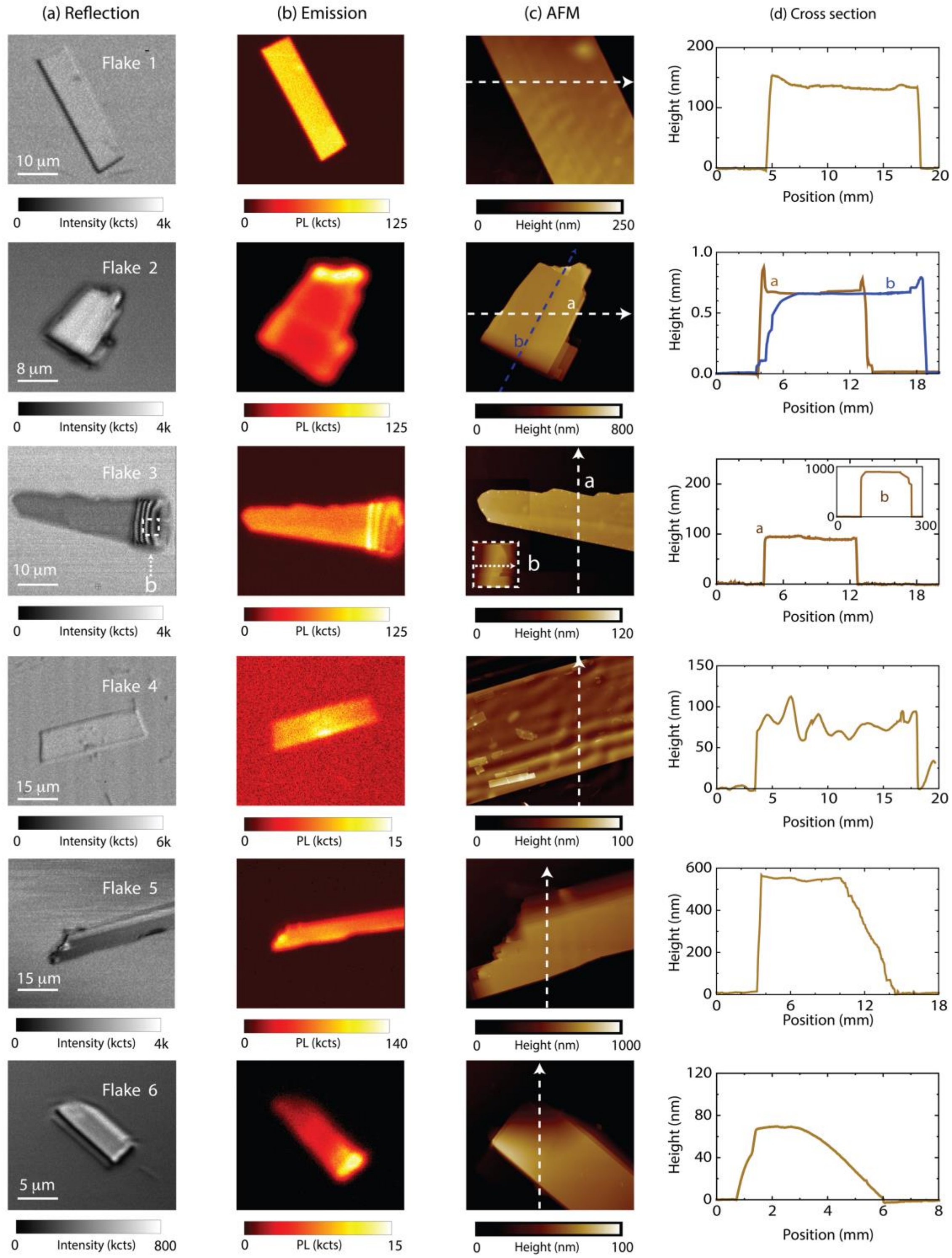

Supplementary Figure 4: Room-temperature characterization of Er-implanted flakes in the main text. For each flake (rows 1–6): (a) reflected-light micrograph; (b) confocal telecom-band PL map (980-nm excitation; integration 1.50–1.60 μm); (c) AFM topography; (d) height line-profiles taken along the dashed lines in (c) (for Flake 2 and Flake 3, two orthogonal cuts "a" and "b" are shown). PL generally increases with thickness; bright ridges at some edges reflect geometry-dependent out-coupling.

II.c Optical spectroscopy and lifetime measurements

Optical spectra were recorded with a Princeton Instruments SpectraPro 2300i equipped with an InGaAs array and a 600 g/mm, 1600-nm blaze grating optimized for the 1.5-µm band. We first locate the emitting region by confocal imaging, then hold the 980-nm focus on the selected spot, collimate the collected light, and feed it to the spectrometer. Unless noted, each spectrum was integrated for 30 min. Time-resolved photoluminescence (PL) measurements were performed using a pulsed 980-nm diode laser driven by an iC-Haus Eval HB controller and triggered via a PulseBlaster (SpinCore). The sample was excited with a 200-µs laser pulse, after which the PL was recorded using a gated superconducting nanowire single-photon detector (SNSPD) with 50-µs time binning. The fluorescence decay profile was reconstructed by sweeping the delay time over a 10-ms interval.

III Extended data sets

III.a Field-dependent Er PL

The interpretation of the temperature-dependent measurements presented in the main text relies on the assumption that the Er optical response is governed primarily by the local magnetic field experienced by the ion. We therefore first examine the direct response of the Er emission to externally applied magnetic fields. Supplementary Fig. 5 compares telecom- band PL maps of two H-dose flakes with $B = 0$ and $B = 0.3$ T approximately colinear with the magnetic intermediate a-axis. In both cases, we find that the presence of magnetic field reduces the PL intensity by about 20%—both under ambient and cryogenic conditions—a dependence that makes Er a practical reporter of magnetic response.

Considering the strong magnetic dipole component of erbium's telecom transitions, one possible mechanism could involve a reduction of the dipole oscillator strength due to Zeeman mixing[13]. In this scenario, the magnetic field at the Er site perturbs crystal-field levels in a way that reduces the magnetic-dipole radiative rate; assuming the magnetic field leaves the non-radiative decay channels unaffected, the latter leads to longer emitter lifetimes and reduced quantum efficiency —i.e., less PL—as experimentally observed.

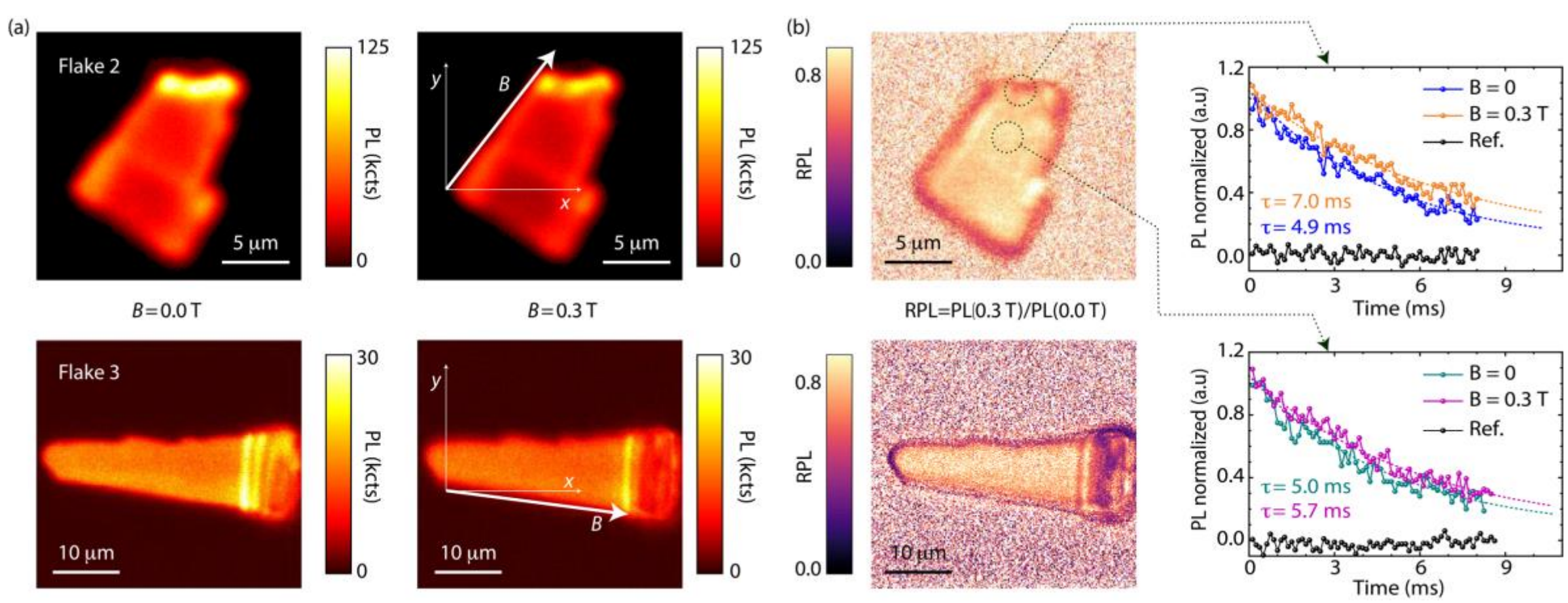

Supplementary Figure 5: Magnetic-field–induced quenching of Er^{3+} photoluminescence in Er:CrSBr. (a) Telecom-band PL maps from two implanted Flakes (2, 3) at $B = 0$ and with an in-plane field $B = 0.3$ T parallel to the a-axis. In both flakes the field reduces the overall PL intensity. (b) Ratio images $\mathrm{RPL} = \mathrm{PL}(0.3\mathrm{T})/\mathrm{PL}(0\mathrm{T})$ highlighting spatially nonuniform quenching. In Flake 2 the suppression is strongest near the perimeter; in Flake 3 it extends to selected bright regions. The edge- or region-selective response is not universal but recurs across samples. (Right-hand side inserts) PL lifetime with and without magnetic field as measured near the top edge of Flake 2 (top) and within its bulk area (bottom); dashed lines indicate exponential fits. Ref: Reference signal in the absence of optical excitation. All data at room temperature and PL integrates emission above 1500 nm.

As shown in the right-hand side insets of Supplementary Fig. 5b, PL dimming is accompanied by longer excited state lifetimes, an anticorrelation we find invariably throughout our experiments. Assuming the non-radiative channels remain unchanged, this combined dependence points to a reduction of the Er radiative rate in the presence of an external field. Note that, similar to the PL intensity, the lifetime change can also be amplified by photonic effects, as seen when comparing the bulk and near-edge responses.

The results in Supplementary Fig. 6 expand on the above observations. We image Flake 3 while toggling a static field between $B = 0$ and 0.3 T and form ratio maps $\mathrm{RPL} = \mathrm{PL}(0.3\mathrm{T})/\mathrm{PL}(0\mathrm{T})$. The response is highly directional: An out-of-plane field ($B \parallel c$, the hardest magnetic axis) produces no discernible change, whereas in-plane fields dim the PL. Moreover, the dimming is largest for $B \parallel a$ and weaker for the orthogonal in-plane orientation. Time-resolved traces (not shown for brevity) confirm the anti-correlation highlighted above, namely, where PL decreases most, the lifetime τ grows most. The latter implies a reduction of the effective radiative rate rather than the creation of a fast non-radiative channel. Further, polarization-resolved data (see next sub-section below) show that under field the emission and excitation axes do not rotate; only the amplitude changes.

The spatial pattern of the dimming is typically non-uniform. While interiors show a relatively gentle, homogeneous reduction, edges and thickness steps frequently display enhanced suppression in the RPL maps. This edge sensitivity suggests that part of the transduction is photonic: The field alters CrSBr's magneto-optical response (birefringence/dichroism), which — in concert with local geometry — modifies the local density of optical states (LDOS) and the fraction of Er emission that couples into radiative vs guided/leaky modes. Edges and thickness gradients are where the LDOS is most field-susceptible, explaining the stronger local effect. We warn that the effect is not universal among flakes, vanishing in $\lesssim$50 nm thick flakes.

This said, a purely photonic explanation cannot account for the temperature-dependent PL/lifetime trends: To reproduce the dip near T_N and the recovery deep in the AFM phase, the Er center must sense the local magnetic field itself, i.e., an "atomic" effect is mandatory. In this regime the relevant interaction

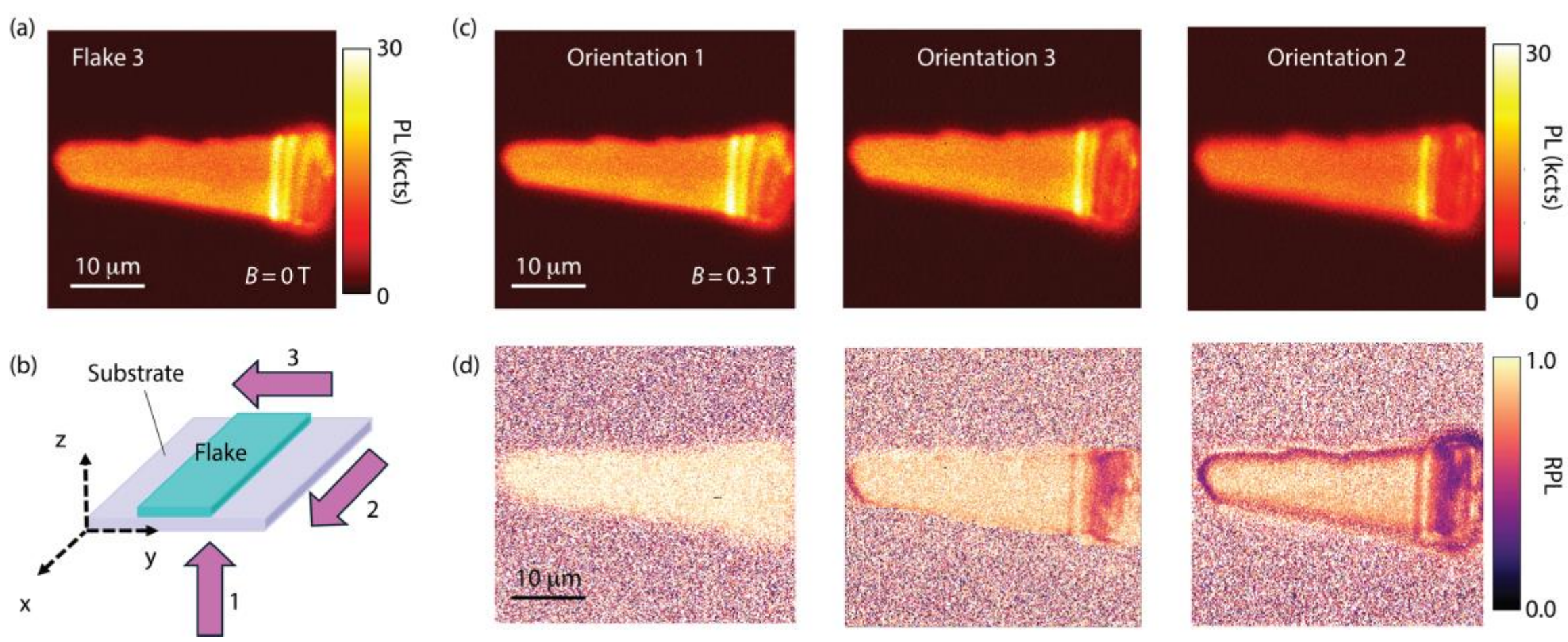

Supplementary Figure 6: In- and out-of-plane field orientation dependence of Er^{3+} PL in CrSBr. (a) Confocal telecom-band PL map of Flake 3 at $B = 0$ T (980-nm excitation). (b) Schematic of the three field orientations used (arrows 1–3) with respect to the flake/substrate axes. (c) PL maps of Flake 3 at $B = 0.3$ T for orientations 1, 3, and 2 (left to right). The PL response is strongly directional: an out-of-plane field (B∥c) produces negligible change in brightness, whereas in-plane fields reduce the PL. The dimming is largest when B is aligned with the a-axis (intermediate magnetic axis) and weaker for the orthogonal in-plane orientation. (d) Ratio maps $\mathrm{RPL} = \mathrm{PL}(0.3\ \mathrm{T})/\mathrm{PL}(0\ \mathrm{T})$ visualize this anisotropy and also reveal that the suppression is often edge-enhanced, while the interior shows a smaller, more uniform reduction. All measurements at room temperature.

volume is nanoscopic (set by the Er crystal-field wavefunction and exchange fields over ~1–2 nm), so the readout reflects how well the two AFM sublattices cancel at the Er site and how much uncompensated moment resides in its immediate neighborhood.

At room temperature—well above T_N—we cannot yet pin down why the dimming is strongest for $B \parallel a$. A conservative reading is that the anisotropy of the system sets the directionality, which could arise from any combination of anisotropic paramagnetic susceptibility, anisotropic magneto-optical response, and anisotropy of the Er crystal field/g-tensor—all different faces of the same underlying directional preference. Separately, given the evidence in Main Fig. 2 for residual AFM correlations well above T_N, it is also plausible that a field along a cants these short-range moments more effectively, enhancing the local net field at Er; this mechanism is well-motivated at low T and could persist at room temperature to the extent that such correlations survive.

III.b Excitation and emission polarization

We first map the excitation dipole by rotating the pump polarization at $B = 0.0$ T, $T = 300$ K (Supplementary Fig. 7a). The integrated telecom PL follows an offset–cosine with a clear maximum when the pump aligns with the flake's in-plane a-axis, establishing that Er absorption is strongly linearly polarized. Fixing the pump at this optimum, we then rotate the analyzer (Supplementary Fig. 7b). Both a 1500-nm long-pass and a 1540-nm band-pass yield high-contrast, two-lobed polar plots with a stable analyzer axis, i.e., linearly polarized emission with little wavelength dependence across the dominant Er lines.

Applying an in-plane field $B = 0.3$ T reduces the amplitude of the polar plots but does not rotate either the excitation or emission axes (Supplementary Figs. 7c, 7d). The effect is temperature-robust (seen at 300

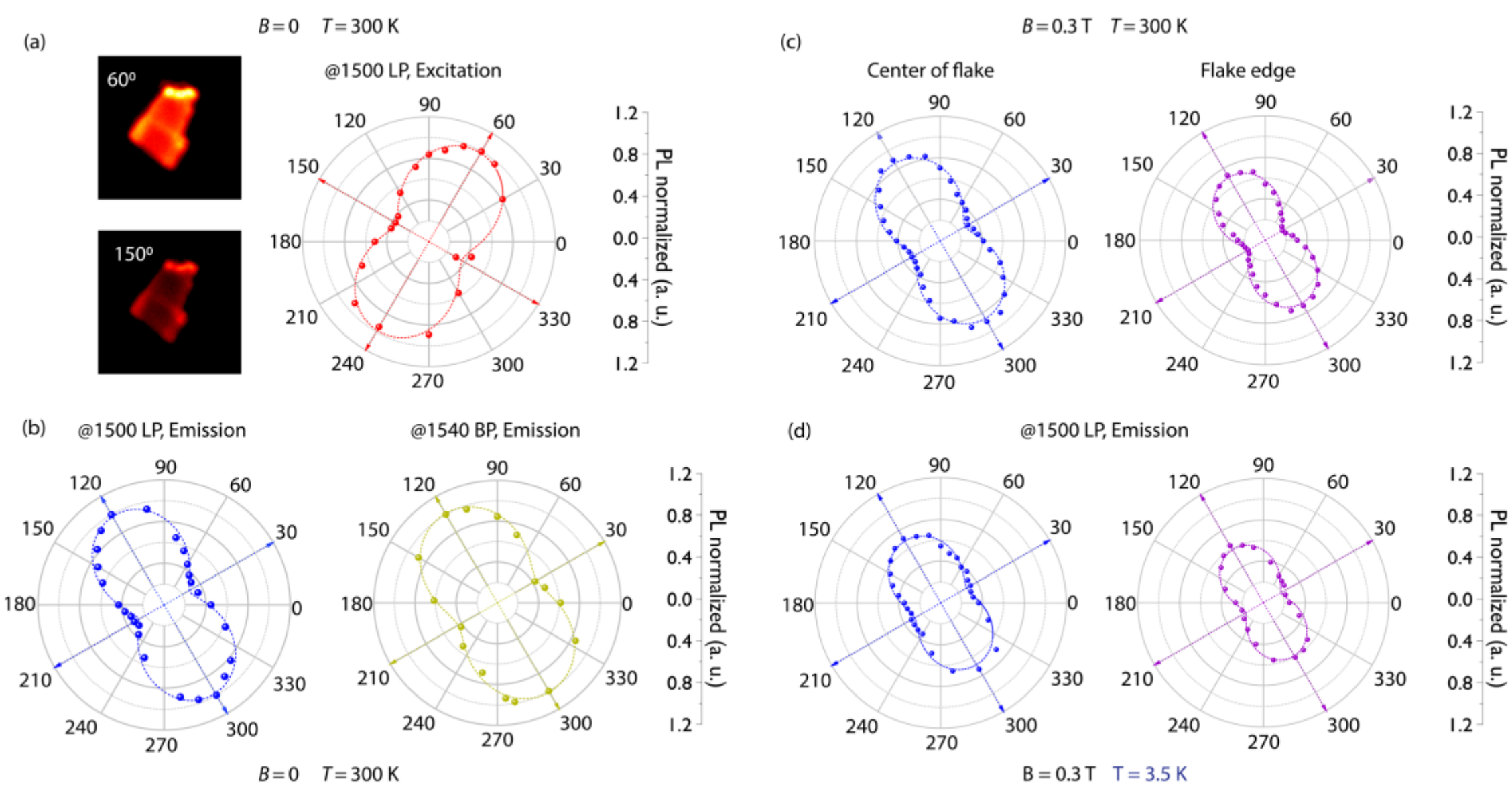

Supplementary Figure 7: Polarization behavior of Er^{3+} emission in CrSBr. (a) Excitation-angle series at $B = 0$, $T = 300$ K: confocal PL maps (left) and corresponding polar plots (right, 1500-nm long-pass) while rotating the pump linear polarization. PL is maximized when the pump is aligned with the flake's in-plane a-axis. (b) Emission polarization with excitation fixed at the optimal angle from (a): Integrated PL versus analyzer angle for a 1500-nm long-pass (left) and a 1540-nm band-pass (right), revealing strongly linearly polarized emission. (c) Effect of an in-plane field ($B = 0.3$ T) at $T = 300$ K: Analyzer-angle dependence recorded separately from the flake center (left) and edge (right) using a 1500-nm long-pass. The field reduces the overall amplitude but leaves the polarization axis unchanged; edge regions show a larger modulation. (d) Same measurement as (c) at $T = 3.5$ K. Curves are normalized to the maximum PL at 300 K and $B = 0$ T. Across all conditions we observe amplitude changes without rotation of the excitation or emission dipoles. In (c) and (d), the field points along the a-axis.

K and 3.5 K) and position dependent: Edge regions show a larger field-induced suppression than the flake interior (consistent with the observations in Fig. 2 of the main text). Combined, these observations indicate that the field primarily changes the magnitude of the radiative channel (or its out-coupling) rather than reorienting the optical dipole. The stronger edge response points to a photonic transduction component—edges and thickness gradients are where the LDOS/out-coupling are most sensitive—while the axis stability constrains purely atomic mechanisms to those that modulate oscillator strength without dipole rotation. This polarization behavior is therefore consistent with the two-stage picture developed in the main text: a local magnetic perturbation at the Er site, whose readout is amplified by the local photonic environment, especially near edges.

III.c PL dependence on flake thickness (extends Figs. 2b–c in the main text)

In the main text we showed that the PL–temperature curves depend strongly on thickness: The thin (~70 nm) flake (Fig. 2b) exhibits a dip near T_{N} with modest recovery of PL deep in the AFM phase, whereas the thick (~600 nm) flake (Fig. 2c) shows a similar dip but a much stronger low-T recovery. Supplementary Fig. 8 demonstrates the same trend within one sample (Flake 7) containing adjacent regions of different thickness. AFM confirms a clear height difference between Region 1 (thicker) and Region 2 (thinner); the corresponding PL–T traces reproduce the behavior above: The thinner region recovers comparatively more weakly below T_{N}, while the thicker region exhibits a larger PL rebound; note that the thickness difference within Flake 7 is smaller than seen within the flakes in the main text, consistent with a milder contrast between curves.

Panel (d) extends this comparison across other flakes and quantifies this trend by plotting the normalized PL contrast $\Delta\mathrm{PL} = \big(\mathrm{PL}(T) - \mathrm{PL}(132\ \mathrm{K})\big)/\mathrm{PL}(300\ \mathrm{K})$ at $T = 3.5$ K and 300 K versus thickness; both low-T and room-T contrasts rise rapidly from ~70–150 nm and then saturate for thicker slabs. Combined, the intra-flake control (a–c) and cross-flake statistics (d) rule out flake-to-flake variability and show that the temperature response is systematically thickness-dependent. This finding seems to suggest that thicker slabs attain better sublattice cancellation (smaller net local field at the Er site).

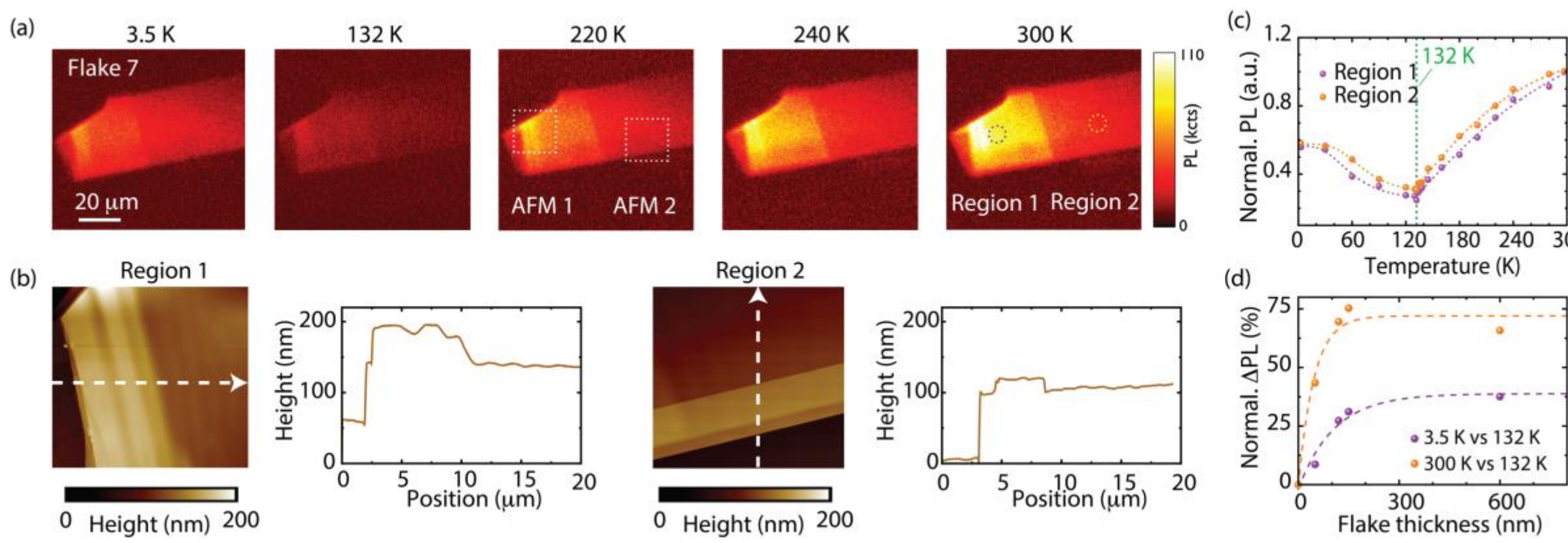

Supplementary Figure 8: Thickness-dependent PL–temperature response within a single flake. (a) Telecom-band PL maps of an Er-implanted CrSBr flake recorded while sweeping temperature (selected frames). Two areas of different thickness are indicated (Region 1: thicker; Region 2: thinner). (b) AFM topography and line profiles from the dashed boxes in (a) reveal the step in thickness between Region 1 and Region 2 (left/right panels, respectively). (c) Flake-integrated PL versus temperature extracted separately from the two regions under warm-up starting from 3.5 K. The thicker area (purple) reproduces the "thick-flake" behavior of the main text (deeper minimum and stronger recovery below T_N), whereas the thinner area (orange) follows the "thin-flake" trend (shallower minimum and more gradual evolution). The vertical dashed line marks the bulk Néel temperature $T_N \approx 132$ K. (d) Normalized percentage difference $\Delta\mathrm{PL} = \big(\mathrm{PL}(T) - \mathrm{PL}(132\ \mathrm{K})\big)/\mathrm{PL}(300\ \mathrm{K})$ for temperatures $T = 3.5$ and 300 K (purple and orange circles, respectively) as derived from the data in (d) (and similar data sets for other flakes); dashed lines are guides to the eye. All data acquired with 980-nm excitation.

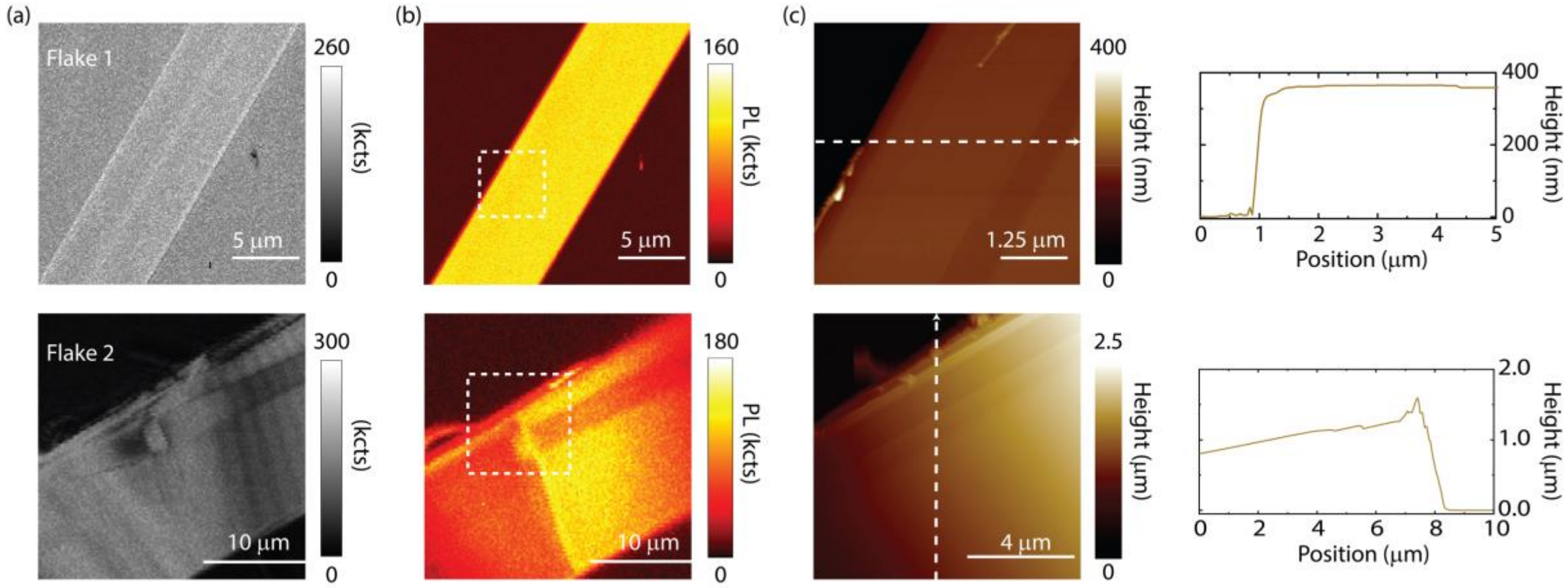

Supplementary Figure 9: Characterization of Er:CrSBr flakes implanted at reduced fluence. Representative CrSBr flakes implanted with an Er fluence of 5×10^{11} ions cm^{-2} (20× lower than the samples used in the main text). (a) Reflected-light optical micrographs. (b) Confocal telecom-band PL maps acquired under 980-nm excitation. Dashed squares indicate the regions examined by atomic force microscopy (AFM). (c) AFM topography of the corresponding regions, with height profiles along the dashed lines shown in the right column. All observations at room temperature.

III.d Low-fluence implantation control

One concern with ion-implanted probes is that the implantation process itself may introduce lattice disorder and thereby perturb the intrinsic magnetic properties of the host material. To assess whether the magneto-optical response reported in the main text is governed by implantation-induced damage, we repeated the principal measurements using CrSBr flakes implanted with a substantially reduced Er fluence of 5×10^{11} ions cm^{-2}, twenty times lower than the 1×10^{13} ions cm^{-2} employed throughout the main study.

Representative flakes are presented in Supplementary Fig. 9. Despite the lower implantation fluence, both samples exhibit readily detectable telecom-band photoluminescence under 980-nm excitation. Reflected-light microscopy, confocal PL imaging, and AFM confirm well-defined flakes with optical and structural quality comparable to those investigated in the main text.

More remarkably, after accounting for differences in flake thickness, the integrated PL brightness is found to be comparable to that of the higher-dose samples (see, e.g., Supplementary Fig. 3b). This observation indicates that the detected emission does not simply scale with the number of implanted ions. Instead, it suggests that the telecom PL is dominated by a subset of optically active Er centers occupying favorable local environments, while a significant fraction of the implanted ions remain optically inactive or contribute only weakly to the measured signal. Although the microscopic structure of these emitting centers remains to be established, a plausible scenario is that they correspond to Er ions occupying well-defined substitutional or near-substitutional lattice configurations.

Temperature-dependent spectroscopy and integrated PL measurements are summarized in Supplementary Fig. 10. Despite the large reduction in implantation fluence, we observe the same qualitative behavior reported in the main text: The Er emission lines narrow dramatically upon cooling, becoming resolution-limited at low temperature (Panel (a)); the integrated PL exhibits a pronounced minimum near the Néel temperature; and the emission recovers upon further cooling into the antiferromagnetic phase (Panel (b)). Within experimental uncertainty, the overall temperature evolution is unchanged, including the PL hysteresis observed upon undergoing a thermal cycle and the dependence with thickness highlighted in the main text (Fig. 2; see also Supplementary Fig. 8). Also notice that annealing at 400 °C produces only minor changes in the temperature dependence of the PL, indicating that the observed magneto-optical response is not governed by metastable implantation defects.

Combined, these observations argue against implantation-induced damage as the dominant origin of

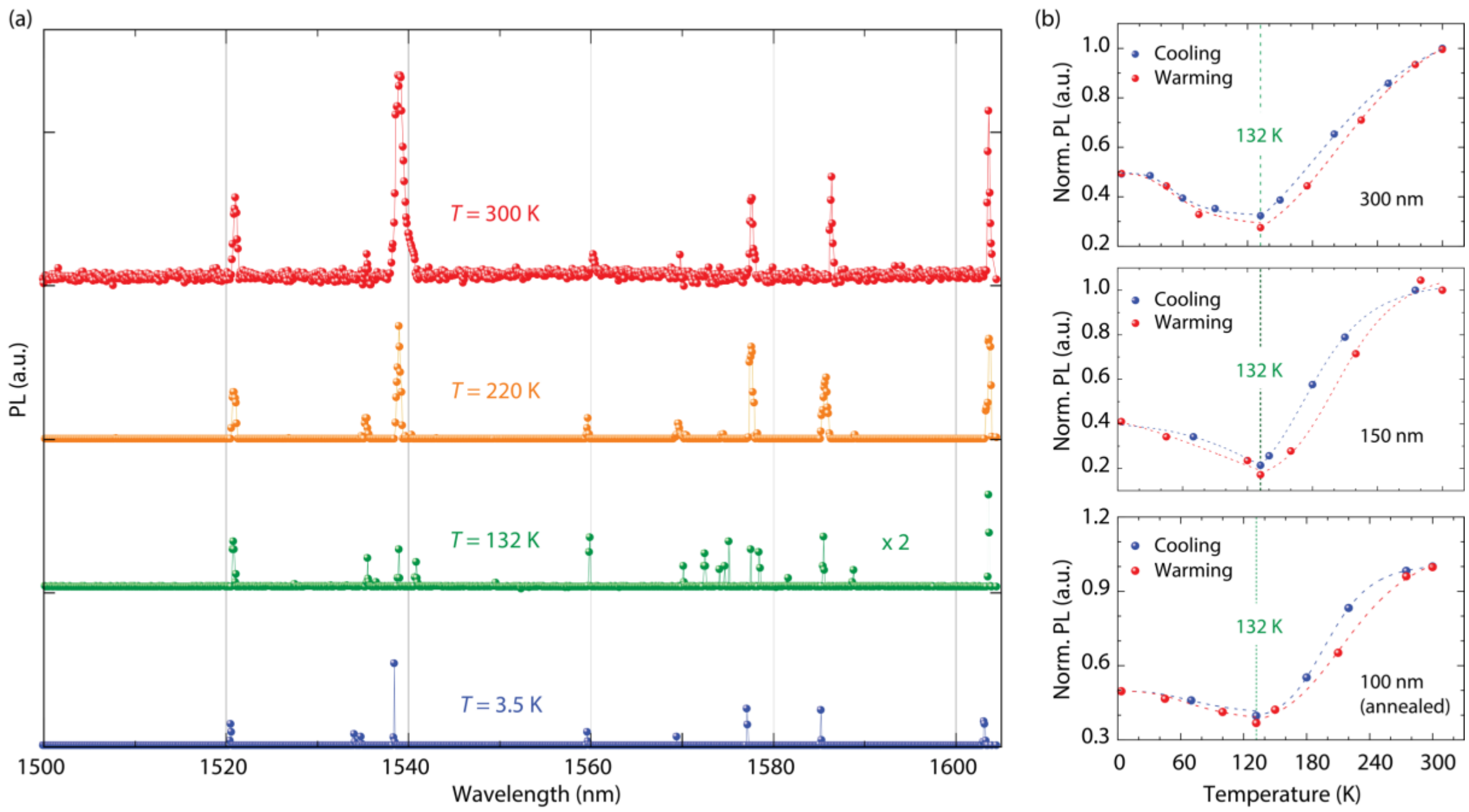

Supplementary Figure 10: Temperature-dependent optical response of CrSBr implanted with a reduced Er fluence. Measurements on CrSBr flakes implanted with 5×10^{11} Er ions cm^{-2} (20× lower than the fluence used in the main text). (a) Temperature-dependent telecom photoluminescence spectra acquired at selected temperatures. The spectra exhibit the same qualitative evolution observed for the higher-fluence samples, including the emergence of sharp, well-resolved Er resonances at low temperature and the redistribution of spectral weight near the Néel transition. (b) Normalized integrated PL intensity as a function of temperature for representative flakes of different thicknesses (300 and 150 nm) and for a 100-nm flake following annealing at 400 °C. All samples display a pronounced PL minimum near the Néel temperature ($T_N \approx 132$ K), while the overall temperature dependence varies systematically with flake thickness but remains essentially unchanged after annealing.

the reported magneto-optical response. Moreover, the combination of comparable PL brightness and resolution-limited low-temperature emission suggests that the detected signal originates from a relatively homogeneous sub-ensemble of Er centers experiencing similar crystal fields. Additional experimental and theoretical work will be required to fully identify the microscopic structure of these centers.

IV First-principles calculations

IV.a Methods

Our DFT calculations are performed using VASP[14], with exchange-correlation interactions described via the semi-local generalized-gradient approximation (GGA) parameterized by Perdew, Burke and Ernzerhof (PBE)[15]. Core electrons are treated via the projector-augmented wave (PAW) pseudopotentials[16]. We include the erbium 4*f* electrons in the valence. A kinetic energy cutoff of 400 eV is chosen for the plane-wave basis expansion. All atomic structures are constructed from a bulk CrSBr unit-cell, with lattice parameters fixed to those from experiments[17,18]. Defect structures are generated using a 3×3×4 CrSBr supercell (containing 216 atoms in the pristine case), which is constructed from the lattice-fixed unit-cell. For erbium-related defects, a single erbium impurity is considered in each case. The erbium interstitial is initialized with the erbium atom at the largest-space site between layers, i.e., at the center of four intralayer bromine atoms with one (intralayer) sulfur atom above and one additional (interlayer) bromine atom below erbium. All calculations are employed at the Γ-point, with tight convergence criteria for forces (10^{-3} eV/Å) and electronic iterations (10^{-8}) to ensure well-converged structures and wave functions, respectively. Since the CrSBr lattice parameters are fixed to those from experiments, we have neglected dispersion corrections

within DFT in all our calculations and initialize the magnetic moments in the anti-ferromagnetic (AFM) ordering. For Hubbard (DFT+*U*) corrections, we employ the Liechtenstein[19] scheme, taking *U* and *J* to be 4.0 (7.0) and 1.0 (1.0) for chromium *d* (erbium *f*) orbitals, respectively, all in units of eV[20,21]. No onsite corrections were applied to bromine and sulfur atoms.

IV.b Correlated effective model

Following Refs. [22] and [23], we resort to a semi-empirical Slater-Hund's correlated model to describe the many-body states and optical transitions of Er^{3+} in CrSBr. The model construction and diagonalization are performed using the TRIQS software library[24]. Compared to our previous work on Er:WS_2, the current model neglects electric components of the TDMs and constructs the symmetrized CF solely from fits to the experimental optical transition lines, assuming a C_{2v} site symmetry. The rest of the parametrization and model construction is identical to Refs. [22] and [23].

References

[1] A. Scheie, M.E. Ziebel, D.G. Chica, Y.J. Bae, X. Wang, A.I. Kolesnikov, X. Zhu, X. Roy, "Spin waves and magnetic exchange Hamiltonian in CrSBr", *Adv. Sci.* **9**, 2202467 (2022).

[2] K. Wittmaack, "Reliability of a popular simulation code for predicting sputtering yields of solids and ranges of low-energy ions", *J. Appl. Phys.* ***96** (2004) 2632.*

[3] P.L. Grande, F.C. Zawislak, D. Fink, M. Behar, "Range parameters study of medium-heavy ions implanted into light substrates", Nucl. Instr. Meth, **61** (1991).

[4] S. Moll, Y. Zhang, Z. Zhu, P.D. Edmondson, F. Namavar, W.J. Weber, "Comparison between simulated and experimental Au-ion profiles implanted in nanocrystalline ceria", *Nucl. Instr. Meth. Phys. Res. B* **307**, 93 (2013).

[5] Y. Zhang, I.T. Bae, K. Sun, C.M. Wang, M. Ishimaru, Z. Zhu, W. Jiang, W.J. Weber, "Damage profile and ion distribution of slow heavy ions in compounds", *J. Appl. Phys*. **105**, 104901 (2009).

[6] A. Polman, "Erbium implanted thin film photonic materials", *J. Appl. Phys.* ***82***, 1 (1997).

[7] K. Eder, V. Bhatia, J. Qu, B. Van Leer, M. Dutka, J.M. Cairney, "A multi-ion plasma FIB study: Determining ion implantation depths of Xe, N, O and Ar in tungsten via atom probe tomography", *Ultramicrosc*. **228**, 113334, 2021.

[8] C. Borschel, C. Ronning, "Ion beam irradiation of nanostructures A 3D Monte Carlo simulation code", *Nucl. Instrum. Methods Phys. Res. B* **269**, 2133 (2011).

[9] W. Möller, "TRI3DYN, "Collisional computer simulation of the dynamic evolution of 3-dimensional nanostructures under ion irradiation"", *Nucl. Instr. Meth. Phys. Res. B* **322**, 23 (2014).

[10] G. García-Arellano, G.I. López Morales, Z. Shotan, R. Kumar, B. Murdin, C. Dreyer, C.A. Meriles, "Erbium-implanted WS_2 flakes with room-temperature photon emission at telecom wavelengths", *Nano Lett.* **25**, 9070 (2025).

[11] S.W. Song, J. Klein, K. Mosina, Z. Sofer, D. Sedmidubský, J. Dong, K. Chen, F.M. Ross, R. Jaramillo, "Magnetic secondary phases in the annealed van der Waals antiferromagnet CrSBr", *J. Phys. Chem. C* **129**, 19002 (2025).

[12] M. Weile, S. Grytsiuk, A. Penn, D.G. Chica, X. Roy, K. Mosina, Z. Sofer, J. Schiøtz, S. Helveg, M. Rösner, F.M. Ross, J. Klein, "Defect complexes in CrSBr revealed through electron microscopy", *Phys. Rev. X* **15**, 021080 (2025).

[13] G. García-Arellano, G.I. López Morales, J. Flick, Cyrus, E. Dreyer, C.A. Meriles, "Magnetically tunable telecom emission from Er^{3+} ions in layered WS_2", arXiv: 2603.17118 (2026).

[14] G. Kresse, J. Furthmüller, "Efficient iterative schemes for ab initio total-energy calculations using a plane-wave basis set", *Phys. Rev. B* **54**, 11169 (1996).

[15] J. P. Perdew, K. Burke, M. Ernzerhof, "Generalized gradient approximation made simple", *Phys. Rev. Lett.* **77**, 3865 (1996), erratum *Phys. Rev. Lett.* **78**, 1396 (1997).

[16] P. E. Blöch, "Generalized gradient approximation made simple", *Phys. Rev. B* **50**, 17953 (1994).

[17] S. A. López-Paz, Z. Guguchia, V. Y. Pomjakushin, C. Witteveen, A. Cervellino, H. Luetkens, N. Casati, A. F. Morpurgo, F. O. von Rohr, "Dynamic magnetic crossover at the origin of the hidden-order in van der Waals antiferromagnet CrSBr", *Nat. Commun.* **13**, 4745 (2022).

[18] M. Bianchi, S. Acharya, F. Dirnberger, J. Klein, D. Pashov, K. Mosina, Z. Sofer, A. N. Rudenko, M. I. Katsnelson M. Rösner, M. van Schilfgaarde, P. Hofmann, "Paramagnetic electronic structure of CrSBr: Comparison between ab initio GW theory and angle-resolved photoemission spectroscopy", *Phys. Rev. B.* **115**, 235107 (2023).

[19] A. I. Liechtenstein, V. I. Anisimov, J. Zaanen, "Density-functional theory and strong interactions: Orbital ordering in Mott-Hubbard insulators", *Phys. Rev. B* **52**, R5467 (1995).

[20] X. Bo, F. Li, X. Xu, X. Wan, Y. Pu, "Calculated magnetic exchange interactions in the van der Waals layered magnet CrSBr", *New J. Phys.* **25**, 013026(2023).

[21] B. Liu, Y. Wang, Y. Liu, Y. Xu, X. Chen, H. Song, Y. Bi, H. Liu, H. Song, "Comparative study of first-principles approaches for effective Coulomb interaction strength U_{eff} between localized *f*-electrons: Lanthanide metals as an example", *J. Chem. Phys.* **158**, 084108 (2023).

[22] G. García-Arellano, G. I. López Morales, Z. Shotan, R. Kumar, B. Murdin, C. Dreyer, C. A. Meriles, "Erbium-implanted WS_2 flakes with room-temperature photon emission at telecom wavelengths", *Nano Lett.* **25**, 9070 (2025).

[23] G. García-Arellano, G. I. López Morales, J. Flick, C. E. Dreyer, C. A. Meriles, "Magnetically tunable telecom emission from Er^{3+} ions in layered WS_2", *arXiv*:2603.17118v2 (2026).

[24] O. Parcollet, M. Ferrero, T. Ayral, H. Hafermann, I. Krivenko, L. Messio, P. Seth, "TRIQS: a toolbox for research on interacting quantum systems", *Comp. Phys. Comm.* **196**, 398 (2015).
